# Virus–Host Interplay Between Poly (ADP-Ribose) Polymerase 1 and Oncogenic Gammaherpesviruses

**DOI:** 10.3389/fmicb.2021.811671

**Published:** 2022-01-14

**Authors:** Woo-Chang Chung, Moon Jung Song

**Affiliations:** Virus-Host Interactions Laboratory, Department of Biotechnology, College of Life Sciences and Biotechnology, Korea University, Seoul, South Korea

**Keywords:** poly (ADP-ribose) polymerase 1 (PARP1), gammaherpesvirus replication, Epstein-Barr virus (EBV), Kaposi’s sarcoma-associated herpesvirus (KSHV), murine gammaherpesvirus 68 (MHV-68), virus-host interaction, ADP-ribosyltransferase diphtheria-toxin-like 1 (ARTD1)

## Abstract

The gammaherpesviruses, include the Epstein–Barr virus, Kaposi’s sarcoma-associated herpesvirus, and murine gammaherpesvirus 68. They establish latent infection in the B lymphocytes and are associated with various lymphoproliferative diseases and tumors. The poly (ADP-ribose) polymerase-1 (PARP1), also called ADP-ribosyltransferase diphtheria-toxin-like 1 (ARTD1) is a nuclear enzyme that catalyzes the transfer of the ADP-ribose moiety to its target proteins and participates in important cellular activities, such as the DNA-damage response, cell death, transcription, chromatin remodeling, and inflammation. In gammaherpesvirus infection, PARP1 acts as a key regulator of the virus life cycle: lytic replication and latency. These viruses also develop various strategies to regulate PARP1, facilitating their replication. This review summarizes the roles of PARP1 in the viral life cycle as well as the viral modulation of host PARP1 activity and discusses the implications. Understanding the interactions between the PARP1 and oncogenic gammaherpesviruses may lead to the identification of effective therapeutic targets for the associated diseases.

## Introduction

Post-translational modifications are important molecular mechanisms through which a cell regulates the diverse functions of proteins in various biological processes. Among the various cellular post-translational modifications, ADP-ribosylation constitutes a major modification. It involves the covalent attachment of the ADP-ribose unit from nicotinamide adenine dinucleotide (NAD +) onto a target protein. Depending on the acceptor proteins or catalyzing enzymes, ADP-ribosylation may involve the addition of a single unit or a polymer of ADP ribose units. The main proteins catalyzing ADP-ribosylation are PAR polymerases (PARPs) (Gupte et al., 2017). To date, 18 PARPs have been identified in the human genome, based on the conserved PARP motif (Amé et al., 2004; Jubin et al., 2016). Among these, PARP1 is the most abundant protein and has been widely studied as a major PARylating enzyme (Bai, 2015). PARP1 is involved in several cellular events, such as DNA repair, transcription, DNA replication, chromatin remodeling, energy metabolism, and cell death via PARylating target proteins (Kim et al., 2005; Ko and Ren, 2011; Gibson and Kraus, 2012; Luo and Kraus, 2012). Moreover, PARP1 is critical in viral infections, including the retroviruses, herpesviruses, influenza virus, hepatitis B virus, and chikungunya virus (Kameoka et al., 1999, 2005; Dandri et al., 2002; Zhang et al., 2002; Ko and Ren, 2011; Grady et al., 2012; Bueno et al., 2013; Rom et al., 2015; Na et al., 2016; Xia et al., 2020).

The human gammaherpesviruses such as the Epstein–Barr virus (EBV) and Kaposi’s sarcoma-associated herpesvirus (KSHV), are important pathogens associated with various tumors and proliferative diseases (Arvin et al., 2007; Jha et al., 2016). The murine gammaherpesvirus 68 (MHV-68) is genetically and biologically related to the human gammaherpesviruses. MHV-68 is extensively studied for elucidating the virus-host interactions and pathogenesis of gammaherpesviruses (Speck and Virgin, 1999; Virgin and Speck, 1999; Nash et al., 2001). These oncogenic herpesviruses display host cell tropisms, infecting epithelial, endothelial, fibroblastic, and lymphoblastic cells for their replication and establishing latency in lymphoid tissues, mainly T or B cells, through which they establish a persistent life-long infection in the hosts (Roizman et al., 1981). Interestingly, various host proteins have been identified to regulate gammaherpesvirus infections (Ye et al., 2011; Lee et al., 2012; Murata, 2014; Niller et al., 2014). Among these, PARP1 plays a key role in regulating the life cycle of the gammaherpesvirus. In this review, we have summarized the roles of PARP1 in the latent and lytic infection phases of the gammaherpesviruses, such as KSHV and EBV, and discussed how these oncogenic herpesviruses modulate PARP1 to promote their replication in the host. Furthermore, this review discusses the implications and the future perspectives of the activities of PARP1 on modulating these viruses.

### PARP1: Biological Functions

**FIGURE 1:**
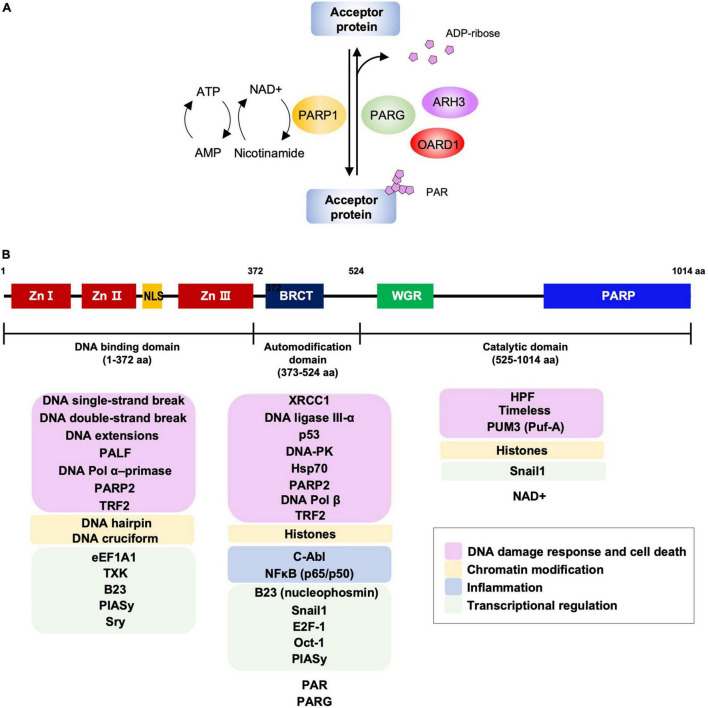
PARP1-mediated PARylation and PARP1 structural domains with interacting partners. **(A)** The PARylation reaction by PARP1. The activated PARP1 hydrolyses NAD^+^ and catalyzes the transfer of ADP-ribose moiety onto target residues of the acceptor protein (PARylation). The PARylated proteins are involved in various biological processes, including DNA repair, cell death, chromatin remodeling, inflammation, and transcription. PAR chains from the PARylated acceptor proteins are rapidly catabolized by PAR glycohydrolase (PARG), ADP-ribosylhydrolase 3 (ARH3), and O-acyl-ADP-ribose deacylase 1 (OARD1). NAD^+^, nicotinamide adenine dinucleotide. **(B)** Schematic representation of PARP1 domains and interaction partners. PARP1 contains three domains; the N-terminal DNA binding domain that has three zinc finger motifs with DNA binding activity and an NLS that is responsible for the localization of PARP1 into the nucleus; the auto modification domain that has a BRCT domain and is responsible for its auto-PARylation; the catalytic domain that contains a homologous PARP family motif and catalyzes PARylation of the target proteins. According to the studies using PARP1 domain mutants, PARP1 interacting partners for each domain are listed and marked for the associated cellular activities. Zn I, Zn II, and Zn III: zinc finger motifs; NLS, nuclear localization signal; BRCT, BRCA1 C-terminus motif; WGR, tryptophan-glycine-arginine-rich domain; PARP, PARP activity domain.

PARPs or ADP-ribosyltransferase diphtheria-toxin-like (ARTDs) are enzymes that catalyze the transfer of the ADP-ribose unit from NAD^+^ to specific residues in the target proteins, resulting in the addition of ADP-ribose polymers, a process termed as PARylation (Kawaichi et al., 1980; Ogata et al., 1980a,b). Therefore, the availability of the NAD^+^ pool in the cells is a key factor for the activity of PARPs. PARP1 is the founding member among the 18 human PARPs. This highly conserved eukaryotic nuclear protein is known to play a role in DNA damage response, chromatin modification, transcriptional regulation, inflammation, and cell death (Gibson and Kraus, 2012). PARP1 mediates approximately 90% of the cellular PARylation in response to DNA damage (Bai, 2015). PARP1 is responsible for the majority of cellular PARP activity, followed by PARP2, while the activities of other PARPs seem to be negligible in comparison (Bai, 2015). The PARylated proteins can be reversely hydrolyzed by PAR glycohydrolase (PARG), ADP-ribosylhydrolase 3 (ARH3), or O-acyl-ADP-ribose deacylase 1 (OARD1) to remove the PAR chain from the target protein and catabolize ADP-ribose (Oka et al., 1984, 2006; Davidovic et al., 2001; Rosenthal et al., 2013; Sharifi et al., 2013). A schematic diagram for addition or removal of PAR to an acceptor protein is shown in Figure 1A.

PARP1 contains three domains, an N-terminal DNA-binding domain (DBD), a central auto-modification domain (AD), and a C-terminal catalytic domain (CAT) (Alvarez-Gonzalez et al., 1999; Affar et al., 2001; Kim et al., 2005; Figure 1B). The DBD contains three zinc-finger motifs (ZnFI, ZnFII, and ZnFIII) and a nuclear localization sequence and are responsible for recognizing the DNA breaks such as a single-strand break (SSB) or double-strand break (DSB) (Caldecott et al., 1996; Langelier et al., 2008, 2011, 2012). The AD includes the BRCA1 C-terminus (BRCT) motif, flanked by lysine and glutamate residues as auto-PARylation sites (Altmeyer et al., 2009; Tao et al., 2009). The BRCT motif mediates interactions with other proteins involved in various cellular pathways (Masson et al., 1998; Beernink et al., 2005; Cuneo et al., 2011). The CAT domain contains a helical WGR domain and an ADP-ribosyltransferase (ART) domain that is conserved in the PARP family proteins (Hottiger et al., 2010). It is responsible for catalyzing the addition of ADP-ribose units onto target proteins and their subsequent elongation and branching (Mendoza-Alvarez and Alvarez-Gonzalez, 1993). PARP1 has been reported to interact with and/or PARylate various cellular factors including PARP1 itself to modulate their functions (Gibson and Kraus, 2012; Ciccarone et al., 2017; Kamaletdinova et al., 2019). Although domain-mapping studies for protein-protein interactions using domain mutants of PARP1 have not been done for all the interacting proteins, representative binding partners for its individual domains are listed and marked with the associated cellular activities in Figure 1B (Kraus and Lis, 2003; Glover et al., 2004; Strosznajder et al., 2005; Li et al., 2006; Kanno et al., 2007; Hassa and Hottiger, 2008; Kotoglou et al., 2009; Krishnakumar and Kraus, 2010; Chang et al., 2011; Rodríguez et al., 2011; Gibson and Kraus, 2012; Ko and Ren, 2012; Mangerich and Bürkle, 2012; Xie et al., 2015; Bonfiglio et al., 2017; Ray Chaudhuri and Nussenzweig, 2017; Alemasova and Lavrik, 2019; Kamaletdinova et al., 2019; Ke et al., 2019; Fehr et al., 2020; Demény and Virág, 2021).

### PARP1 and Viral Infections

PARP1 plays important roles in infections of viruses with DNA genome. For example, PARP1 binds to the hepatitis B virus (HBV) core promoter and enhances viral gene transcription and HBV replication (Ko and Ren, 2011). A study on PARP1 inhibitors suggested that PARP1 activity may limit the integration of the HBV DNA (Dandri et al., 2002). As an essential factor for HBV replication, HBx interacts with PARP1 and inhibits the recruitment of the DNA repair machinery to the damaged DNA sites, thereby promoting hepatocarcinogenesis (Na et al., 2016). In vaccina virus infection, PARP1 plays a role in NK cell migration to the site of infection and promotes CCL2 production (Shou et al., 2019). Herpes simplex virus 1 degrades the poly (ADP-ribose) glycohydrolase (PARG) using the viral E3-ligase, ICP0 and activates PARP1 to facilitate virus replication (Grady et al., 2012).

PARP1 activity also regulates the replication of many RNA viruses. In human immunodeficiency virus 1 (HIV-1) infection, PARP1 activation increases the integration of the HIV-1 genome into the host chromosome (Ha et al., 2001; Kameoka et al., 2005). PARP-1 activity also increased long terminal repeat (LTR)-mediated viral gene transcription (Kameoka et al., 1999; Rom et al., 2015). In contrast, there are reports showing a negative role of PARP-1 in viral gene transcription (Parent et al., 2005; Bueno et al., 2013). For human T-lymphotropic virus 1 (HTLV-1), PARP1 activates transcription of Tax binding elements (Zhang et al., 2002). In Influenza A virus, hemagglutinin (HA) interacts with PARP1 and degrades type I interferon receptor (IFNAR), thereby enhancing virus replication (Xia et al., 2020). PARP1 interacts with the nucleocapsid (N) protein of porcine reproductive and respiratory syndrome virus (PRRSV) and its activity promotes the virus replication (Liu et al., 2015). Sindbis virus (SINV), belonging to the *Alphavirus*, activates PARP1 during infection (Ubol et al., 1996; Nargi-Aizenman et al., 2002). PARP1 is found in the SINV replication complex and interacts with nsP3, suggesting its positive role in the replication of SINV (Park and Griffin, 2009). Overall effects of PARP1 on regulation of viral replication can be either proviral or antiviral, while the effects appear to be both in HIV-1 infection.

## PARP1 in EBV Infection

EBV, a member of *Lymphocryptovirus* genus, is the first discovered human oncovirus that is associated with Burkitt’s lymphoma, infectious mononucleosis, Hodgkin’s disease, nasopharyngeal carcinoma, gastric carcinoma, and various other lymphomas (Rickinson, 2014). It has a worldwide prevalence with over 95% of the adults being seropositive for EBV (Luzuriaga and Sullivan, 2010). As it spreads primarily through saliva, most of the individuals are infected during infancy and early childhood asymptomatically or with non-specific symptoms.

### Life Cycle of EBV

EBV is known to spread mainly through body fluids, such as saliva, blood, and semen, while organ transplantation is also a potential transmission route (Chang et al., 2009). After primary infection, the virus remains latent in the individuals for the rest of their lives. EBV establishes latency mainly in the B lymphocytes and is reactivated intermittently by the induction of lytic replication (Dugan et al., 2019). A subset of viral genes is expressed to maintain latency that causes proliferation of the infected B lymphocytes. EBV-infected cells exhibit three latency programs, latency I, II, or III. Latency I restricts the expression to that of the most latent genes, while latency III involves the expression of a full repertoire of the known latent genes. During latency III, the EBV nuclear antigens (EBNAs 1, 2, 3A, 3B, 3C, and LP), latent membrane proteins (LMPs 1, 2A, and 2B), and viral non-coding RNAs (EBERs, miRNAs, and BARTs) are expressed, establishing the lymphoblastoid cell lines (Rickinson, 2002). The selective pressure from the EBV-specific cytotoxic lymphocytes leads to the transition from latency III to more restricted forms of latency. Latency II is characterized by the lack of *EBNA2* and *EBNA3* expression but exhibits the expression of other latent genes (Price and Luftig, 2015). *EBNA1* is the only viral gene expressed in latency I. In latency 0, the viral genome persists in the host cells without viral gene expression, which is associated with infection in the non-dividing memory B-cells (Dugan et al., 2019). When EBV reactivation is induced, the immediate-early (IE) genes, *BZLF1* and *BRLF1* are transcribed to produce Zta (also called ZEBRA or EB1) and Rta (also called R), respectively. After the synthesis of these transcription factors, the downstream lytic genes, including their promoters, are activated, resulting in a full cascade of lytic gene expression. The early (E) genes encode proteins required for DNA replication and metabolism, such as the viral DNA polymerase, viral DNA primase, processivity factor, and thymidine kinase. Following viral DNA replication, the late (L) genes are transcribed for the synthesis of structural proteins to assemble the virion particles (Young et al., 2007).

### Roles of PARP1 in EBV Infection

**FIGURE 2:**
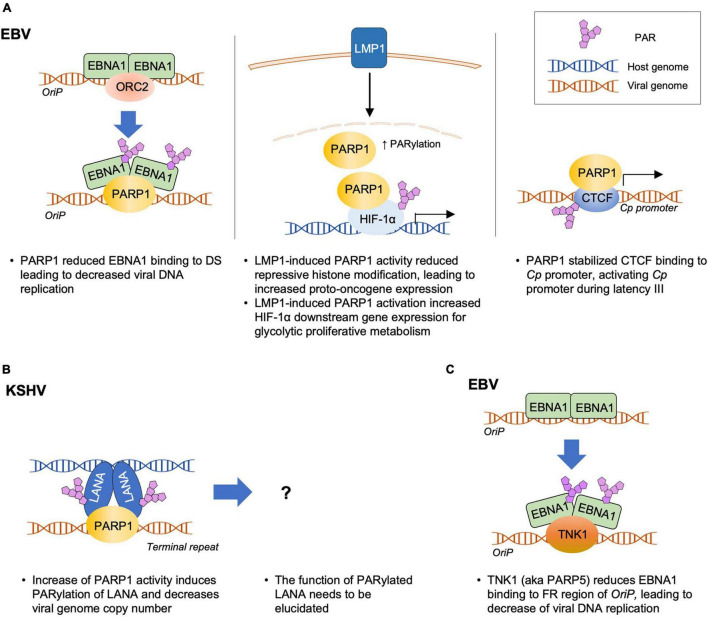
Molecular Interactions of PARP1 or TNK1 with gammaherpesviruses during latency. PARP1 modulates latent infection of gammaherpesviruses, EBV **(A)** or KSHV **(B)**. In turn, LMP1 induces PARP1 activity, contributing to the tumorigenesis of EBV. **(C)** TNK1 (aka PARP5) downregulates the EBNA1-dependent *OriP* replication. The detailed mechanisms are explained in the text and summarized in Table 1.

PARP1 is involved in the regulation of EBV latent infection (Figure 2A). During latency, the replication of viral DNA depends on the interaction between EBNA1 and origin of plasmid replication (*OriP*) in the virus (Kennedy and Sugden, 2003; Lindner and Sugden, 2007). PARP1 was identified as one of the cellular factors that bind to the dyad symmetry (DS) elements of the *OriP*, the site for EBNA1 binding (Deng et al., 2002, 2005; Tempera et al., 2010). Treatment of D98 cells with the PARP inhibitors, such as niacinamide (NA) and 3-aminobenzamide (3-ABA), resulted in a significant increase in the *OriP* plasmid maintenance after 3 weeks of treatment (Deng et al., 2002, 2005; Tempera et al., 2010). In contrast, a ribonucleotide reductase inhibitor, hydroxyurea (HU), that increases PARP1 activity, caused a substantial loss of the *OriP* plasmids in these cells, which was consistent with a previous report that HU accelerated the loss of EBV genomes from Akata cells (Srinivas et al., 1998; Deng et al., 2002, 2005; Tempera et al., 2010). Mechanistically, PARP1 was shown to induce PARylation of EBNA1, which reduced the binding affinity of EBNA1 to the DS of *OriP* (Tempera et al., 2010). Studies on PARP1 knockdown and treatment with PARP1 inhibitors further suggest that PARP1 suppresses EBNA1 binding and the recruitment of origin recognition complex 2 (ORC2) onto *OriP*, leading to a reduction in the replication of viral DNA during latency (Tempera et al., 2010). In addition, PARP1 is colocalized with the CCCTC-binding factor (CTCF), a host insulator protein that binds to specific sites throughout the EBV genome including the latency promoter Cp (Lupey-Green et al., 2018). PARP1 PARylates CTCF and studies involving PARP1 inhibitors showed that PARP1 stabilizes CTCF binding and maintains the open chromatin structure of the EBV genome at the active *Cp* promoter during type III latency (Lupey-Green et al., 2018). Taken together, these results suggest that PARP1 and PARylation of EBNA1 and CTCF are important mechanisms regulating the viral episome during EBV latency.

A recent study indicated the involvement of PARP1 in the lytic cycle of the EBV replication. PARP1 binds to the *BZLF1* promoter in the type I and type III latently infected B-cells and prevents binding of Zta to its promoter for autoregulation, thereby limiting the EBV reactivation (Lupey-Green et al., 2017). In another study, treatment with the PARP1 inhibitor, 3-ABA, during EBV reactivation increased early antigen expression (Mattiussi et al., 2007). However, PARP1 inhibition increased LMP1 and EBNA2 expression, but decreased the expression of BFRF1, a nuclear egress protein, reducing the overall virion production in the culture media (Mattiussi et al., 2007). These results suggest that PARP1 enzymatic activity may play a role in the progression of the EBV lytic cycle.

### Viral Modulation of PARP1 in EBV Infection

In EBV-associated tumorigenicity, LMP1 employs PARP1 for epigenetic control. LMP1 expressed in type II and III latency programs is the major transforming protein that is critical for EBV-induced B-cell transformation and modulate several processes, such as differentiation, cell migration and survival, and tumorigenicity (Li and Chang, 2003; Shair et al., 2012; Liu et al., 2018). Martin et al. (2016) reported that LMP1 hijacks PARP1 to enhance the cellular PARylation level and thus enhances cellular transformation. They showed that PARP1 suppressed the expression of enhancer of zeste 2 polycomb repressive complex 2 subunits (EZH2), a histone methyltransferase, and a catalytic component of the initiation complex, polycomb repressive complex 2 (PRC2); this, in turn, reduced the level of the repressive histone marker, trimethylation of lysine 27 on histone H3 (H3K27me3), and induced the expression of LMP1-related protooncogenes *c-Fos* and *EGR1* (Martin et al., 2016). Hulse et al. (2018) showed that LMP1 activated PARP1 to increase the hypoxia-inducible factor 1-alpha (HIF-1α)-dependent gene expression. In latently infected cells, LMP1-activated PARP1 acts as a coactivator of HIF-1α, forming the PARP1–PARylated–HIF-1α complex, as depicted in Figure 2A. This complex bound to the promoters of genes downstream of *HIF-1*α and increased their expression, resulting in altered cell metabolism and a switch from mitochondrial respiration to a glycolytic “Warburg” metabolism (Hulse et al., 2018).

**FIGURE 3:**
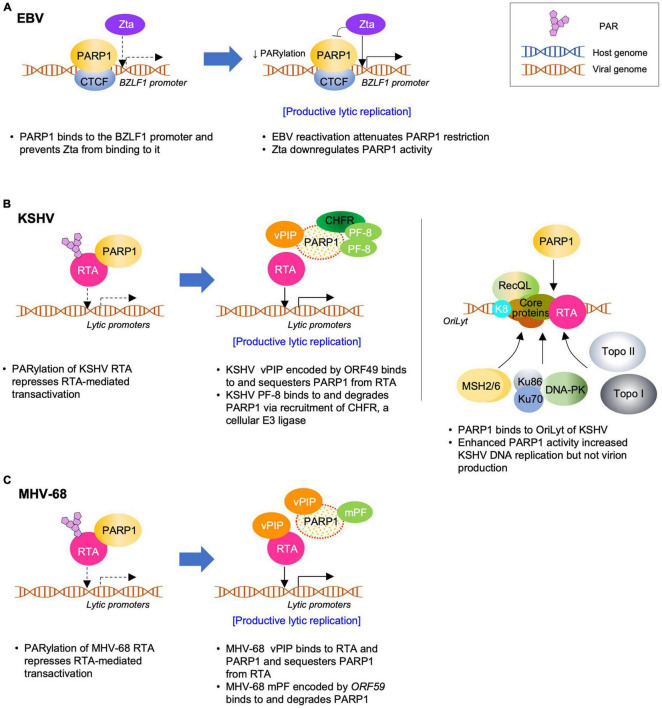
Molecular Interactions between PARP1 and gammaherpesviruses during lytic replication. PARP1 regulates lytic replication of EBV (A), KSHV (B), or MHV-68 (C). The viruses also, in turn, modulate PARP1 via sequestering or degrading PARP1 to promote virus replication. The detailed mechanisms are explained in the text and summarized in Table 1.

EBV suppresses the inhibitory effect of PARP1 to achieve successful lytic replication (Figure 3A). Upon reactivation, PARP1 was downregulated and *BZLF1*-encoded Zta overexpression reduced PARP1 levels to some extent, which contributes to the easy accessibility of *BZLF1* promoter. This makes Zta bind to *BZLF1* promoter and facilitates lytic replication (Lupey-Green et al., 2017). In KSHV and MHV-68, viral processivity factors were shown to downregulate PARP1 in a proteosome-dependent manner. In EBV infection, BMRF1 gene-encoded EA-D primarily acts as a DNA polymerase processivity factor as a component of the viral DNA replication complex to facilitate DNA synthesis (Kiehl and Dorsky, 1991; Tsurumi, 1993; Neuhierl and Delecluse, 2006). It will be intriguing to find out whether EBV EA-D may downregulate PARP1 in a similar way, suggesting a conserved viral mechanism for PARP1 downregulation among gammaherpesviruses.

## PARP1 in KSHV and MHV-68 Infections

After its identification as an infectious agent of Kaposi’s sarcoma (KS), KSHV was confirmed as the eighth member of the human herpesviruses (HHV-8) and classified as a member of the *Rhadinovirus* genus of the gammaherpesvirus subfamily, based on its genome sequence and homology analysis (Chang et al., 1994; McGeoch and Davison, 1999). In addition to KS, KSHV is associated with primary effusion lymphoma (PEL), multicentric Castleman’s disease (MCD), body-cavity-based lymphoma, and other lymphoproliferative diseases (Di Alberti et al., 1997; Rettig et al., 1997; Ganem, 2007). Epidemiology showed that 50% of the adults in sub-Saharan Africa and less than 5% of the adults in northern Europe, North America, and Asia are seropositive for KSHV (Simpson et al., 1996; Calabro et al., 1997; Whitby and Boshoff, 1998). Similar to EBV, saliva is the main transmission route for KSHV, but sexual contact, blood transfusion, and organ transplantation are also considered important routes (Mayama et al., 1998; Corey et al., 2002; Henke-Gendo and Schulz, 2004; Martro et al., 2004). MHV-68 (also known as γHV-68), another member of the *Rhadinovirus* genus, is homologous to EBV and KSHV in terms of genome organization and pathogenesis and is therefore considered an excellent small animal model to study virus-host interactions and viral pathogenesis of human gammaherpesviruses (Simas and Efstathiou, 1998). MHV-68 also provides an amenable genetic system to investigate viral gene functions *in vitro* and *in vivo*.

### Life Cycle of KSHV and MHV-68

After primary infection of the permissive cells, KSHV enters the latent phase and only expresses the latency-associated genes without productive lytic replication. Unlike EBV, KSHV does not induce immortalization of the B-cells nor direct transformation of any infected cells (Myoung and Ganem, 2011). Moreover, there are no diverse latency programs in KSHV, albeit a subset of latent genes may be differentially expressed depending on the cell type. During latency, only a few genes are expressed; these include the *open reading frame (ORF)73* encoding the latency-associated nuclear antigen (LANA or LNA), *ORF72* encoding a viral cyclin D homolog (vCyclin), and *ORF71* encoding a viral Fas-associated protein with death domain-like interleukin-1β-converting enzyme/caspase-8-inhibitory protein (vFLIP), as well as those encoding K12, viral interferon regulatory factors (vIRFs), and viral miRNAs (Cai et al., 2010). LANA is a major latent protein expressed in KSHV-positive malignancies serving as a hallmark for KSHV latency (Rainbow et al., 1997; Gao et al., 1999). It is necessary for DNA replication, maintenance, and segregation of the KSHV episomes during host cell mitosis (Hu et al., 2002; Purushothaman et al., 2016). LANA also acts as a multifunctional nuclear protein, interacting with various cellular proteins involved in tumorigenesis, cellular transcription, and chromatin remodeling (Purushothaman et al., 2016; Wei et al., 2016). When reactivation is induced in the KSHV latently-infected cells, transcription cascade of viral lytic genes activates transcription of the *IE* genes, followed by *E* and *L* genes, leading to the production of infectious virions (Sun et al., 1998; Gradoville et al., 2000; Lukac and Yuan, 2007; Schulz and Chang, 2007; Cai et al., 2010). Notably, the replication and transcription activator (RTA), mainly encoded by *ORF50*, acts as a key switch molecule to induce the expression of lytic genes (Lukac et al., 1998, 1999; Sun et al., 1998; Gradoville et al., 2000; Xu et al., 2005). Several cellular factors including PARP1 regulate the RTA activity either negatively or positively, thereby regulating the virus life cycle (Li et al., 2014; Aneja and Yuan, 2017; Yan et al., 2019).

The life cycle of MHV-68 is similar to that of KSHV in many aspects with some discrepancies. *De novo* infection of MHV-68 results in robust lytic replication in the fibroblasts and epithelial cells (Wu et al., 2000; Rochford et al., 2001). MHV-68 RTA, mainly encoded by *ORF50*, is also essential for activating the cascade of downstream lytic gene expression and inducing lytic replication (Liu et al., 2000; Wu et al., 2000). Moreover, MHV-68 LANA, a homolog of the KSHV LANA, plays an important role in establishing and maintaining viral latency (Fowler et al., 2003; Moorman et al., 2003; Habison et al., 2012).

### Roles of PARP1 in KSHV and MHV-68 Infections

PARP1 affects the latent infection of KSHV (Figure 2B). Together with other known replication factors, such as ORC2, CDC6, and Mcm7, PARP1 was identified as a cellular factor that binds to the terminal repeat (TR) of the KSHV genome (Ohsaki et al., 2004). Although PARP1 was colocalized with LANA and induced its PARylation, PARP1 binding to a specific region within the TR was independent of LANA, which leaves the function of PARylated LANA to be elucidated. Nevertheless, NA (a chemical inhibitor of PARP1) treatment increased the viral genome copy number in BC-3 cells, whereas HU (a PARP1 activator) treatment decreased it, suggesting that PARP1 may play a role in maintaining KSHV latency. PARP1 and LANA interaction was also confirmed using the affinity purification method using the N-terminus of LANA (Barbera et al., 2006a). Although Ku70, Ku80, and PARP1 were found to be interacting partners in this study, neither of these factors have been shown to mediate LANA chromosome association (Barbera et al., 2006a). However, the binding of PARP1 to the TR might play a role in genome circularization and latent genome maintenance associated with LANA. Further studies are needed to identify this role (Barbera et al., 2006b).

PARP1 is also an important cellular factor that negatively regulates lytic replication of KSHV (Figure 3B). PARP1 suppresses the KSHV RTA activity by directly binding to and PARylating RTA (Gwack et al., 2003). RTA PARylation decreases the RTA-mediated transcriptional activity by inhibiting the recruitment of RTA to the promoters of lytic genes. Gwack et al. (2003) showed that PARP1 and Ste20-like kinase hKFC interacted with the serine/threonine-rich region of RTA. These two factors synergistically enhanced their interactions with RTA as well as post-translational modifications of RTA, such as PARylation and phosphorylation, thereby acting as strong repressors of the RTA activity on lytic gene expression (Gwack et al., 2003). Using mutant RTA constructs, Ko et al. (2012) showed that the Thr-366 and Thr-367 residues of RTA formed the primary motif for O-GlcNAcylation *in vivo*, which plays a role in the recruitment of PARP1 to RTA. In contrast, PARP1 reportedly plays a positive role in regulating DNA replication during the lytic phase. Using DNA affinity purification, Wang et al. (2008) identified topoisomerases (Topo) I and II, MSH2/6, RecQL, DNA-PK, Ku86/70, scaffold attachment factor A (SAF-A), and PARP1 as KSHV *oriLyt*-bound proteins. Inhibition of PARP1 using chemical inhibitors (3-ABA and NA) resulted in decreased *oriLyt*-dependent DNA replication, whereas HU increased PARP1 activity and DNA replication, suggesting a positive role for PARP1 during the lytic replication of KSHV (Wang et al., 2008). Although the roles of PARP1 in KSHV lytic replication were seemingly inconsistent, Wang et al. (2008) also showed that the overall effect of PARP1 lies in reducing KSHV virion production; the result of lytic replication, suggesting that viral DNA replication may not be the rate-limiting step in virion production and that PARP1-mediated inhibition of RTA activity may be the key step that regulates the KSHV life cycle. Interestingly, the inhibitory effect of PARP1 on virus lytic replication is also conserved in MHV-68 (Gwack et al., 2003). PARP1 reduces the transcriptional activity of MHV-68 RTA and virus lytic replication during *de novo* infection (Figure 3C).

### Viral Modulation of PARP1 in KSHV and MHV-68 Infections

To counteract the inhibitory effect of PARP1 on virus lytic replication, KSHV and/or MHV-68 have been shown to employ two viral proteins during different stages of the life cycle (Figures 3B,C). The MHV-68 *ORF49*-encoded protein, a tegument protein, interacts with and sequesters PARP1 from RTA binding (Noh et al., 2012; Chung et al., 2018). The purified recombinant ORF49 protein from MHV-68 and KSHV interacts directly with PARP1 without any additional cellular factors and is therefore termed viral PARP1 interacting protein (vPIP) (Noh et al., 2012; Chung et al., 2018). vPIP enhances RTA-mediated transactivation by reducing the level of PARylated RTA. Owing to its association with the virion, vPIP is likely to modulate PARP1 function during the early phase of *de novo* infection. Consistent with this hypothesis, the viral growth of a recombinant MHV-68 with transposon or triple stop codons inserted at the ORF49 locus is significantly attenuated *in vitro* and *in vivo*. Furthermore, vPIP residues that are critical for PARP1 interaction were identified based on the vPIP crystal structure (Chung et al., 2018). A recombinant MHV-68 harboring mutations at the three critical residues of vPIP exhibited defective PARP1 interaction and was highly attenuated in viral growth both *in vitro* and *in vivo*, suggesting the significant role of vPIP and PARP1 interaction in derepressing RTA for viral replication.

Other studies have reported that KSHV and MHV-68 downregulate PARP1 in a proteosome-dependent manner during lytic replication (Chung et al., 2015, 2021). KSHV *ORF59* encodes the viral processivity factor, PF-8, which interacts with and degrades PARP1, thereby enhancing the RTA-mediated transactivation, especially on the *RTA* promoter (Chung et al., 2015, 2021). PARP1 degradation is dependent on PF-8 interaction and recruitment of a cellular ubiquitin E3-ligase, checkpoint with FHA and RING finger domains (CHFR) (Chung et al., 2015, 2021). As the viral processivity factor is expressed as an early lytic gene following RTA expression, the PF-8-induced degradation of PARP1 may be important in reducing the inhibitory effect of PARP1 on RTA and further reinforcing the RTA positive feedback to promote lytic replication.

## Other PARPs that Regulate Gammaherpesviruses

In addition to PARP1, other PARPs are reportedly involved in gammaherpesvirus replication. Tankyrase 1 (TNK1; PARP5) uses NAD^+^ to PARylate the acceptors by recognizing the RxxPDG motif (TNKS-binding motif) (Smith et al., 1998; Sbodio and Chi, 2002). TNK1, TNK2 (PARP6), and telomeric repeat binding factor 2 (TRF2) interact with *OriP* in an EBNA1-dependent manner, as determined by the DNA affinity purification assay (Deng et al., 2002). Like PARP1, TNK1 PARylates EBNA1 and downregulates the EBNA1-dependent *OriP* replication (Deng et al., 2005; Tempera et al., 2010), as shown in Figure 2C. Interactome studies have identified interactions between PARPs and viral proteins. A yeast two-hybrid screening for EBV viral proteins with a human cDNA library showed that EBV BRRF1, a homolog of KSHV and MHV-68 vPIP interacts with PARP4 (Calderwood et al., 2007). Furthermore, an interactome study with KSHV ORF libraries using affinity purification/LC-MS has identified PARP2 as an interaction partner of LANA (Davis et al., 2015). However, further studies should be warranted to confirm their genuine interactions and to elucidate the significance of these interactions in the context of virus life cycle.

## Discussion

PARP1 plays a role in various cellular mechanisms, such as DNA damage repair, cell death, proliferation, differentiation, gene transcription, and inflammation (Kim et al., 2005; Ko and Ren, 2011; Gibson and Kraus, 2012; Luo and Kraus, 2012). Additionally, it plays a role in oncogenic gammaherpesvirus infection and modulates the viral life cycle in either a positive or negative manner. Table 1 summarizes the effects of PARP1 on the life cycle of gammaherpesviruses, while Figures 2, 3 show. Although the overall effects of PARP1 on the virus life cycle either promote or inhibit the lytic replication or latency, there are a few discrepancies regarding the roles of PARP1, with reports of both positive and negative effects on the distinct stages of the life cycle of the virus. For example, several studies reported that PARP1 exerts a negative effect on the lytic replication of KSHV (Gwack et al., 2003; Wang et al., 2008; Lupey-Green et al., 2017), while a few reported its positive effect on the lytic replication of KSHV or downregulation of latent genome (Ohsaki et al., 2004; Wang et al., 2008). These discrepancies may be attributed to the differences in the experimental methods employed in individual studies. Moreover, the PARP1 inhibitor, 3-ABA is also a potent inhibitor of apoptosis, which makes it difficult to interpret the contradicting results. In addition, the virus may potentially utilize or overcome the effects of PARP1 depending on the stage of its life cycle. Furthermore, the mode of action of PARP1 can be either dependent or independent of its enzymatic activity, depending on the target proteins or DNA. Therefore, these factors should be considered when attempting to elucidate the roles of PARP1 by thoroughly examining the effects of *PARP1* knockdown or overexpression on virus DNA replication, lytic gene transcription, viral protein expression, and ultimately, virion production.

**TABLE 1:** Summary of PARP1 actions and virus interactions on regulation of gammaherpesvirus life cycle.

Overall outcome	Life cycle	Virus	Effects of PARP1 and virus interactions	References
Positive	Latency	EBV	• LMP1 interacts with PARP1 and enhances the cellular PARylation levels and cellular transformation. • PARP1 suppresses the EZH2 expression, reducing repressive histone modification, and induces the LMP1-related protooncogene expression. • The LMP1-induced PARP1 activation increases the HIF-1α-dependent gene expression. • The PARylated HIF-1α–PARP1 complex binds to promoters of the *HIF-1*α downstream genes, activating their expression and altering the cellular metabolism.	Martin et al., 2016; Hulse et al., 2018
			
			• PARP1 is colocalized with CTCF, stabilizing its binding to latency *Cp* promoter, thereby activating *Cp* promoter during latency III.	Lupey-Green et al., 2018
	
	Lytic infection	EBV	• Treatment with a PARP1 inhibitor increases EA expression, but decreases expression of BFRF1, a nuclear egress protein. • Inhibition of PARP1 activity reduces the overall virion production. • Inhibition of PARP1 activity increases LMP1 and EBNA2 expression.	Mattiussi et al., 2007
			
		KSHV	• PARP1 binds to KSHV *ori-Lyt* DNA. • Increased PARP1 activity enhances the *ori-Lyt* DNA replication.	Wang et al., 2008

Negative	Latency	EBV	• PARP1 binds to the dyad symmetry (DS) element of *OriP*, the site for EBNA1 binding. • PARP1 PARylates EBNA1 and reduces EBNA1 binding to DS, leading to decreased viral DNA replication.	Deng et al., 2002, 2005; Tempera et al., 2010
			
		KSHV	• PARP1 binds to LANA. • PARP1 is recruited to the terminal repeat in the KSHV genome and colocalized with LANA. • Increased PARP activity induces PARylation of LANA and decreases viral genome copy number.	Ohsaki et al., 2004; Barbera et al., 2006a,b
	
	Lytic infection	EBV	• PARP1 binds to the *BZLF1* promoter and prevents Zta from binding to the same promoter. • Zta attenuates the PARylation activity of PARP1.	Lupey-Green et al., 2017
			
		KSHV	• PARP1 directly binds to and PARylates KSHV RTA, repressing the RTA-mediated transcriptional activity by inhibiting the recruitment of RTA to the lytic gene promoters. • KSHV vPIP directly binds to and sequesters PARP1 from RTA. • KSHV lytic replication induces PARP1 degradation. • KSHV PF-8 binds to and degrades PARP1 via recruitment of a cellular ubiquitin E3 ligase, CHFR, which enhances RTA transactivation.	Gwack et al., 2003; Ko et al., 2012; Noh et al., 2012; Chung et al., 2015, 2018, 2021
		
		MHV-68	• PARP1 directly binds to MHV-68 RTA and inhibits its transactivation. • MHV-68 RTA transactivation and lytic replication are enhanced in the PARP1 knockout cells. • MHV-68 vPIP binds and sequesters PARP1 from RTA, thereby promoting RTA-mediated transactivation. • MHV-68 vPIP mutant viruses with transposon insertion or triple stop codons were highly attenuated in viral growth *in vitro* and *in vivo*. • MHV-68 recombinant virus harboring a vPIP mutation defective in PARP1 interaction exhibits severely attenuated viral growth both *in vitro* and *in vivo*. • MHV-68 lytic replication and mPF induces PARP1 degradation.	Gwack et al., 2003; Noh et al., 2012; Chung et al., 2015, 2018
				

As PARP1 activity requires and thereby lowers the cellular NAD^+^ levels, the activity of other NAD^+^-dependent deacetylases, sirtuins (SIRTs) may be affected (Houtkooper et al., 2012). PARP1 activation downregulates SIRT activity accompanied by lowered NAD^+^ level, while SIRT1 activation reduces PARP activity, suggesting that SIRTs and PARPs compete for the cofactor NAD^+^ (Zhang, 2003; Pillai et al., 2005; Kolthur-Seetharam et al., 2006; Bai et al., 2011). SIRT1 inhibition or knockdown reportedly induced KSHV lytic replication, increased active histone H3K4me3 mark, and decreased histone H3K27me3 mark in the RTA promoter. SIRT1 also interacted with RTA, inhibiting its transactivation activity, thereby regulating KSHV lytic replication (Li et al., 2014). Similarly, SIRT6 reportedly bound to KSHV genome and suppressed lytic gene expression (Hu et al., 2019). It will be interesting to study how these two competing factors, SIRTs and PARPs, regulate and balance the cellular NAD^+^ level during virus life cycle and how the gammaherpesviruses modulate these factors for their own benefit.

In conclusion, PARP1 modulates gammaherpesvirus life cycle at lytic replication or latency stage. Interestingly, gammaherpesviruses surmount the inhibitory effects of PARP1 using viral proteins that interact with PARP1 or exploit PARP1 activity for efficient replication. However, the detailed modes of action of PARP1 need to be elucidated under various conditions during virus lytic replication or latency that result in different consequences. Further investigations regarding these aspects will expand our understanding of the interactions between PARP1 and oncogenic gammaherpesviruses, which in turn may lead to identification of effective therapeutic targets for the associated diseases.

## Author Contributions

W-CC and MJS: conceptualization, writing—review and editing, and visualization. MJS: supervision, project administration, and funding acquisition. Both authors have read and agreed to the published version of the manuscript.

## Conflict of Interest

The authors declare that the research was conducted in the absence of any commercial or financial relationships that could be construed as a potential conflict of interest.

## Publisher’s Note

All claims expressed in this article are solely those of the authors and do not necessarily represent those of their affiliated organizations, or those of the publisher, the editors and the reviewers. Any product that may be evaluated in this article, or claim that may be made by its manufacturer, is not guaranteed or endorsed by the publisher.
